# Development of Rice Cake Fortified With Acorn Flour and Inulin Using Superheated Steam Technology: Analysis of Physicochemical, Structural, and Baking Properties

**DOI:** 10.1002/fsn3.4759

**Published:** 2025-01-15

**Authors:** Zohreh Mokhtari, Aman Mohammad Ziaiifar, Mehran Alami, Mahdi Kashaninejad, Sara Aghajanzadeh, Adel Dezyani, Edris Arjeh

**Affiliations:** ^1^ Department of Food Process Engineering Gorgan University of Agricultural Sciences and Natural Resources Gorgan Iran; ^2^ Department of Food Science and Technology Gorgan University of Agricultural Sciences and Natural Resources Gorgan Iran; ^3^ Department of Food Science and Technology, Faculty of Agriculture Urmia University Urmia Iran

**Keywords:** baking process, browning index, condensation, physical properties, superheated steam, volume expansion

## Abstract

Steam injection, especially in a superheated state, increases the rate of heat transfer and improves the quality of the baked products. In this research, different baking methods (forced convention, superheated steam, and superheated steam‐assisted) at different temperatures (140°C, 160°C, 180°C) were applied to produce a new formulated rice cake containing acorn flour and inulin. The findings revealed that the level of moisture inside the oven directly influences the volume of the cake. The cake prepared with supersaturated steam exhibited the lowest volume (67.86 cm^3^ in 25 min, 160°C) and the raw appearance. Whereas, the highest volume (74.87 cm^3^ in 25 min, 160°C) is attained when supersaturated steam is used in conjunction with hot air. The browning index constant in superheated steam‐assisted (0.027 min^−1^) was significantly higher than that in forced convection (0.007 min^−1^) and superheated steam (0.006 min^−1^) treatments. A delayed superheated steam‐assisted method (stream injection after 10 min of baking) improved the crust browning (BI = 120) due to accelerated Maillard reactions and caramelization, while the moist heat can also contribute to a more vibrant and glossy appearance by promoting a smooth, hydrated surface layer. In conclusion, the application of superheated steam and forced convention methods simultaneously during appropriate baking conditions brought better expansion volume, lower hardness and darker crust with suitable moisture content.

## Graphical abstract

Steam injection, especially in a superheated state, increases the rate of heat transfer and improves the quality of the baked products. In this research, different baking methods (forced convention, superheated steam, and superheated steam‐assisted) were applied to produce a new formulated rice cake containing acorn flour and inulin. The findings revealed that delayed superheated steam injection emerged as the most promising technique, offering cakes with balanced moisture content, improved volume, a porous structure, and reduced firmness.

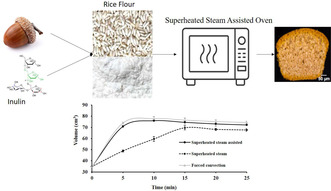

## 1 Introduction

Considering society health perspective (nutritional values and physiological benefits), the consumption of healthy cake containing functional ingredients has been recently of great interest. To produce high‐quality baked products, it is necessary to optimize the baking process like the applied temperature, heating time, and the level of humidity in the baking chamber (Asselman et al. 2007).

In the baking process, the changes in cake properties are mainly controlled by parameters (Cakmak, Mama, and Yilmaz 2021). In new formulated cakes, some changes are not desirable especially during the conventional baking methods such as forced convection. These changes may result in a cake with less acceptance, texture, appearance, taste, and aroma. Different processing conditions are therefore needed to diminish the unwanted changes. It was observed that applying steam‐assisted baking methods can considerably improve the cake properties. In comparison to the conventional baking method, the steam application brings more preservation of the nutritional components and prevents the formation of harmful compounds such as acrylamide (Isleroglu et al. 2012; Sarion, Codină, and Dabija 2021). This is important in the production of newly formulated cakes. Steam injection during the baking process, especially in a superheated state, increases the rate of heat transfer and results in a 10%–20% decrease in energy consumption (Ma et al. 2021).

During the baking process, rising in the temperature of the cake has a key role in product quality. Three periods in temperature variation the central part of the cake were observed: (1) a heating‐up period, in which the temperature almost linearly rises before reaching a plateau, (2) a constant temperature period, characterized by temperature stabilization around 100°C (water evaporation temperature) corresponding to the presence of enough moisture at the central part and (3) a final period, increase in temperature coinciding with crust growth and presenting partially dehydration of the central part (Fehaili, Courel, Rega, and Giampaoli, 2010). Forced convection baking induces rapid evaporation of the water from the dough surface and causes the premature formation of a dry elastic crust. This early formation of crust limits the product expansion so brings an intensive and firm structure (Le‐Bail et al. 2011). As a consequence, the oven spring is restricted in the finished product (Hui et al. 2008).

Steam ovens are often regarded as a healthier alternative to traditional ovens. One of their key benefits is the ability to retain moisture in food during reheating, which minimizes the need for additional fats or oils to maintain moisture. Additionally, cooking with a steam oven helps preserve the natural vitamins and minerals in food, maximizing its nutritional value (Venugopal et al. 2022). Proper processing conditions can also contribute to a lower glycemic index in bread. Zhu (2019) found that variations in the moisture content of Chinese steamed bread compared to hot air‐baked bread were linked to different starch retrogradation patterns, which may affect starch digestibility (Zhu 2019).

Steam baking exhibits distinct effects on the baking process (Cappelli, Lupori, and Cini 2021). Bredariol, de Carvalho, and Vanin (2020) have highlighted differences between bread baked with and without steam (Bredariol, de Carvalho, and Vanin 2020). During steam baking, condensed water on the bread's surface slows heat transfer, reducing the heating rate at the surface. Once the condensation evaporates, heat transfer to the dough's center intensifies, accelerating the heating of the crumb. Steam is commonly introduced in conventional baking to influence the crust's texture or color (Walker 2016). Additionally, steam serves as the primary energy source in the preparation of Chinese‐style steamed bread and a traditional bakery product from Südtirol (northern Italy) known as Schüttelbrot (Mayr et al. 2020).

In steam‐assisted baking strategy, the steam is condensed on the product surface; this phenomenon increases the moisture content as well as the surface plasticity (Altamirano‐Fortoul et al. 2012; Isleroglu et al. 2012), which improves the crust properties. Furthermore, a higher amount of injected steam induces more CO_2_ release from the product resulting in more volume expansions (Fehaili et al. 2010; Ma, Xu, and Xu 2023) resulting in economic benefit especially in cereal based products (Kumari et al. 2015). The volume expansion is controlled by the balance between the internal pressure of the dough and crust resistance (Zhang and Datta 2006). The pressure rise inside the dough results in a compression of the crumb against the crust. According to Bredariol, Spatti, and Vanin (2019) and Dessev et al. (2020), baking parameters such as time, temperature, and steam have an effect on the heating rate and its effects on bread qualities.

To improve marketability and consumer acceptance, cakes and other bakery products can be enriched with acorn (Beltrão Martins et al. 2020) and inulin (Tsatsaragkou et al. 2021) due to their nutritional values and physiological benefits. Acorn is a good source of antioxidants such as gallic and ellagic acids and rich in fiber and electrolytes (Majzoobi et al. 2013; Martins et al. 2022). Inulin, soluble dietary fiber and prebiotic, is a polysaccharide that consists of natural fructose polymer (Bhanja, Sutar, and Mishra 2022). It is used as a sugar and fat replacer in cake formulation. The use of inulin often leads to producing a cake with desirable taste, crispy texture, and expanded volume. It also keeps the cake moist and fresh for a longer period of time (Amini et al. 2022). Steam‐assisted baking results in cookies with comparable quality in terms of spread ratio, bulk density, and textural properties, with a reduced overall baking time and significantly lower acrylamide levels. Similar findings were reported by Bredariol, de Carvalho, and Vanin (2020) for bread. Their study revealed that steam baking produced bread with larger pores compared to bread baked at 160°C and 220°C without steam, while the crumb structure of non‐steam‐baked bread appeared more delicate. The authors suggested this difference might be due to greater expansion during baking (Bredariol, de Carvalho, and Vanin 2020).

This research addresses a significant gap in understanding the specific role of superheated steam in baking, a subject that has been relatively underexplored. By determining the optimal conditions for its application, the study enhances baking science by shedding light on its impact on crust formation, moisture retention, and overall product quality. Furthermore, the findings contribute to advancing energy efficiency and sustainability, providing a novel framework to improve both industrial and artisanal baking methods. The study focused on examining how superheated steam injection influences the physicochemical and structural properties of rice cakes made with acorn flour and inulin, while also analyzing the browning kinetic index to better understand the processes involved in cake formation.

## 2 Material and Methods

### 2.1 Materials

The formulated cake was prepared using rice flour (particle size < 200 μm, Tarom variety) and acorn flour (particle size < 125 μm, *Quercus brantii* ), both of which were procured from the local market in Gorgan, Iran. The inulin used in the study, with a purity of > 99%, was obtained from Food Chem., China. Composition of the rice and acorn flours was showed in Table 1.

**TABLE 1:** Composition of the rice and acorn flours.

Components	Rice flour (%)	Acorn flour (%)
Moisture	10.23	9.31
Protein	9.30	5.15
Fat	0.82	7.00
Fiber	0.20	2.84
Ash	0.45	0.74

### 2.2 Preparation of Cake Batter

To prepare the cake batter, sugar and sunflower oil were first mixed for 4 min at 140 rpm (Sapor mixer, China). Then, eggs were added to the mixture and completely blended (240 rpm, 5 min). After adding (80 rpm, 1 min) the dry ingredients, an aluminum mold (5.6 cm in diameter and 2.81 cm in height) was filled with the prepared cake batter (30 g). Finally, the batter was immediately baked in different conditions (Bennion and Bamford 1997). The amount of each ingredient was listed in Table 2.

**TABLE 2:** Amount of used ingredients in the cake formulations.

Ingredient	Quantity (g)
Rice flour	95 ± 0.08
Acorn flour	5 ± 0.06
Inulin	15 ± 0.09
Egg	72 ± 0.20
Sugar	72 ± 0.07
Vegetable oil	57 ± 0.11
Baking powder	2 ± 0.04
Vanilla	0.5 ± 0.03
Water	30 ± 0.06

### 2.3 Baking Process

**FIGURE 1:**
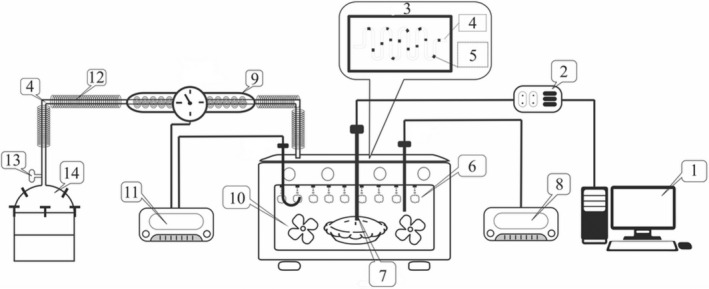
Superheated steam assisted oven: (1) computer, (2) data logger, (3) oven roof, (4) copper tube, (5) pores, (6) superheated steam, (7) product thermocouple, (8) Oven thermocontroller, (9) thermal elements, (10) fan, (11) steam thermocontroller, (12) insulate, (13) Steam outlet valve and (14) steam production unit.

In this study, an electric oven (Leisure, Italy) was equipped with a fan (ensuring forced convention) and a steam injection system (Figure 1). The width, height, and depth of the oven chamber were 65, 35, and 45 cm, respectively. A steam production unit was connected to the oven by an insulated copper tube (1 cm in diameter). Two electrical elements (1000 W) were installed around the tube to produce the superheated steam by controlling its temperature. To achieve a better distribution of the superheated steam inside the oven, pores (1 mm in diameter) were created on the tube. A constant steam injection flow rate (0.7 L/min) was established through the baking process. Two thermo‐controllers were used to keep constant the defined temperature of the superheated steam and circulating oven air. The cakes were baked using three different methods (forced convection, superheated steam, and superheated steam‐assisted) at 140°C, 160°C, and 180°C for 5, 10, 15, 20 and 25 min. Superheated steam and hot air have used simultaneously in the superheated steam‐assisted.

### 2.4 The Cake Properties

#### 2.4.1 The Temperature of Cake Center

During the baking process, the temperature of the central part of the cake was recorded using a data logger (TC‐08, Pico technology Co, UK) equipped with a 1 mm diameter copper‐constantan thermocouple (K‐type).

#### 2.4.2 Moisture Content

The moisture content of the cake was determined by drying the sample up to a constant weight in a convention oven at 105°C (Huang et al. 2021).

#### 2.4.3 Volume Expansion

The volume of the cake was measured according to the rapeseed displacement method (Ma, Xu, and Xu 2023).

#### 2.4.4 Oven Spring

Oven spring refers to the change in the central height of the cake during baking. It was defined based on calculating the difference between the height of the dough and the baked cake (Ayaz et al. 2022). Three replications were done.

#### 2.4.5 Kinetic of Browning Index

Image processing method (ImageJ software, version 1.42e) was used to analyze the captured images using a scanner (HP Scanjet scanner G2710 with a resolution of 600 dpi) to define the color parameters of the cake crust (Turabi, Sumnu, and Sahin 2010). L*, a* and b* were used in the calculation of the browning index (BI) as (Equation 1) (Dadalı, Kılıç Apar, and Özbek 2007).

(1) $$\mathrm{BI}=\frac{\left[100\times \left(\frac{a_{t}+1.79L_{t}}{5.645L_{t}+a_{t}-3.012b_{t}})-0.31\right)\right]}{0.17}$$

where *a*_t_, *b*_t_ and *L*_t_ are a*, b*, and L* color parameters at a definite time (t) of baking, respectively.

To describe the changes in the BI, first‐order semi‐logarithmic kinetic model was used according to (Equation 2) (Aghajanzadeh et al. 2016):

(2) $$\log\left(\frac{A}{A_{0}}\right)=-\mathrm{kt}$$

where *A* and *A*_0_ are respectively final and initial values of the BI. *k* and *t* refer to constant rate (min^−1^) and time of baking (min).

#### 2.4.6 Crumb Hardness

The crumb firmness was determined according to AACC (2000) standard method using a texture analyzer (Model TA‐XT plus, Stable Microsystems, Surrey, England) which was equipped with plunger diameter of 36 mm, plunger speed of 100 mm per minute, load cell 10 kg. For firmness analysis, 1 cm thick cake crumb slices (2 × 2 × 2 cm) were force tested against 50% compression. Crumb firmness were carried out in duplicates for each samples. The average of three hardness values was considered as final crumb hardness (Committee 2000).

### 2.5 Sensory Evaluation

A sensory evaluation of the developed cakes was conducted by 30 untrained tasters who regularly consume cakes. To reduce bias and ensure tasters did not influence each other, a standard sensory evaluation method was used, as outlined by Adanse et al. (2021). The coded samples were presented on clean plastic plates at room temperature. Tasters assessed the acceptability of the samples based on appearance, color, taste, texture, and overall acceptability, using a 5‐point hedonic scale. The scale ranged from 1 (dislike very much), 2 (dislike much), 3 (neither like nor dislike), 4 (like much), to 5 (like very much).

### 2.6 Statistical Analysis

Results were submitted to analysis of variance (ANOVA) with a significance level of *α* = 0.05. The difference between average were analyzed and compared using Duncan's test with a 95% confidence level. All statistical analyses were conducted using the SAS software (version 9.1).

## 3 Result and Discussion

### 3.1 Temperature Profile

**FIGURE 2:**
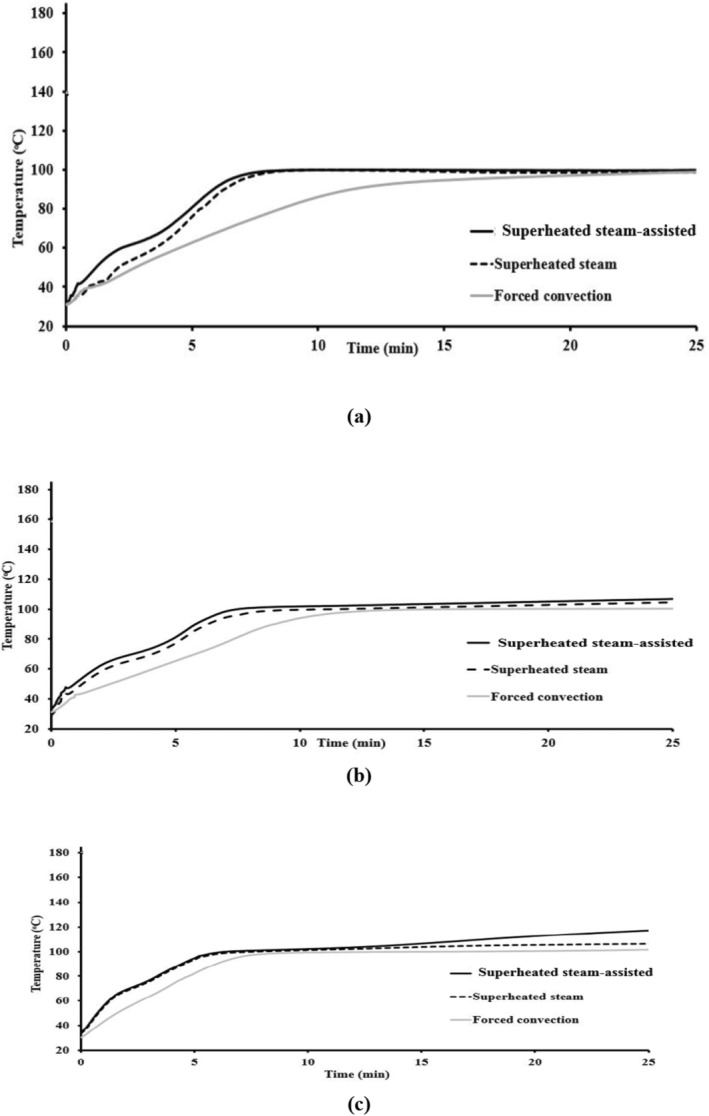
Temperature variation in the central part of the cake during baking at (a) 140°C, (b) 160°C and (c) 180°C using different methods.

Figure 2 shows variation in the temperature of central parts of the cake during the baking process using different methods. A sigmoidal‐shaped curve was observed in all conditions. Unlike the results of other researchers, results showed that applying superheated steam increased considerably (*p* < 0.05) the temperature (Sakin‐Yilmazer et al. 2013). This contradiction may be related to the nature of the used steam (saturated or superheated). Saturated steam injection decreased baking temperature, owing to the heat required to vaporize the liquid water (M Micaela Ureta et al. 2019). It was reported that the thermal history of the inner part of the bakery product was studied to assure complete starch gelatinization and protein denaturation. These features are achieved as the product temperature reaches about 98°C (Ahrné et al. 2007).

In all conditions, baking at a higher temperature increased the center temperature (*p* < 0.05). These results are in agreement with other reports (Sani et al. 2014; María Micaela Ureta, Olivera, and Salvadori 2014). The Highest and lowest raise in the temperature was observed during baking during superheated steam‐assisted and forced convection methods, respectively. Temperature variation became unstable during the superheated steam process as a result of the changes in the heat transfer rate on the surface of the product due to the condensation of vapor or evaporation of moisture. The temperature in the central part of the cake reached rapidly to a constant period due to the high rate of heat transfer and better heat flux distribution. Steam condensation induces enormous heat flux transferred to the sample resulting in a higher heating up rate (Le‐Bail et al. 2011). In the superheated assisted baking process at 180°C, a very short final period and a rapid increase in temperature were observed. In this condition, the temperature tended to reach the baking temperature which caused accelerating the crust formation.

Baking aspects (time, temperature, steam) can be used to modify cake characteristics, in which macromolecular changes are unique only due to different thermal development and mass transfer characteristics (Bredariol, Spatti, and Vanin 2019).

### 3.2 Moisture Content of the Cake

**FIGURE 3:**
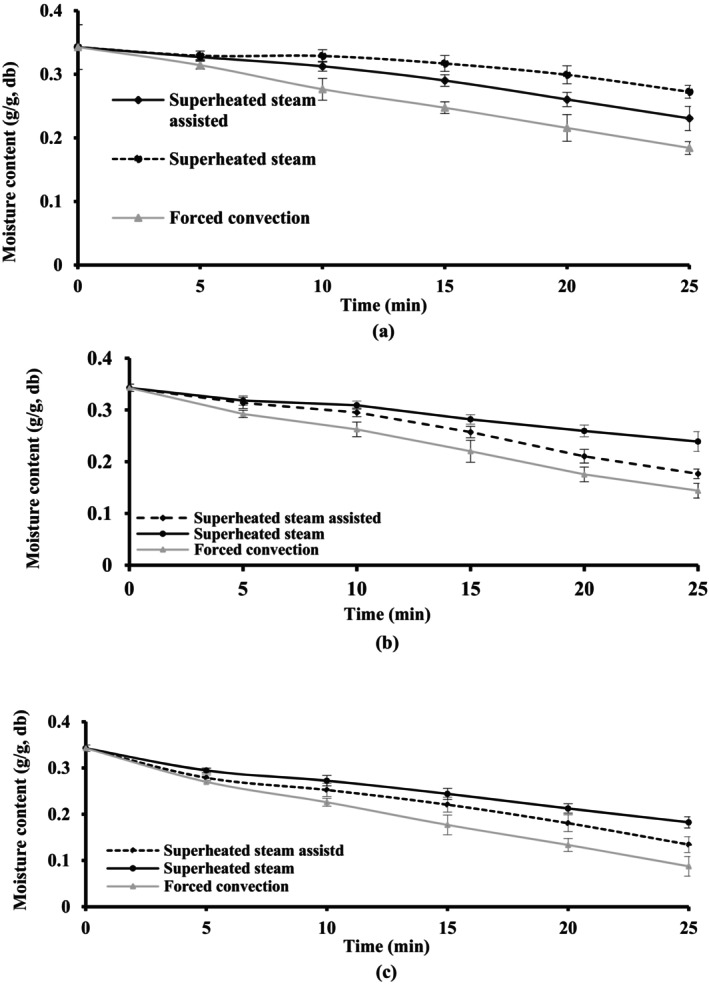
Changes in the moisture content of the cake during baking using different methods: (a) 140°C, (b) 160°C and (c) 180°C.

The changes in moisture content of the baked cakes using different baking methods are represented in Figure 3. In general, the moisture content of the cakes decreased during baking at a higher temperature for a longer time (*p* < 0.05). The baked sample in the forced convection oven had the least moisture content (*p* < 0.05). Under this condition, rapid moisture removal from the dough surface causes the early formation of a dry inelastic shell on the baked cake, oven spring limitation, and surface tearing (Altamirano‐Fortoul et al. 2012; Hui et al. 2008). During the superheated steam baking method, a rise in the temperature of the central part of the cake induced less moisture loss rather than the forced convection one (Figure 3). The highest moisture content was observed in the sample baked by superheated steam (*p* < 0.05). This observation can be explained based on two reasons: (1) Presence of excessive moisture in the oven atmosphere and condensation of water vapor on the product surface (Bredariol, Spatti, and Vanin 2019; Le‐Bail et al. 2011) and (2) A rapid formation of the crust in the early stage of baking (less than 4 min) as a result of rapid surface dehydration. The second reason causes the inhibition of the water migration from the interior parts of the cake to its surface (Purlis and Salvadori 2009). In this way, the crust acts as a barrier to mass transfer towards the oven ambient temperature; hence, it prevents further dehydration of the inner zones, as it was verified in other baked products (Purlis and Salvadori 2009). In this situation, there was no difference between the moisture content of the batter and crumb, and a slight decrease in moisture content was observed at the end of the process.

A balanced moisture content was observed in the superheated steam‐assisted baking method, in this, the moisture content of the oven atmosphere was not excessive because of the presence of the simultaneously forced convection. Therefore, a low amount of condensed steam in the oven chamber created desirable moisture content in comparison to the other methods (Figure 3). The dried and burned crust of the cake at 180°C brought a higher amount of moisture content loss (*p* < 0.05).

**FIGURE 4:**
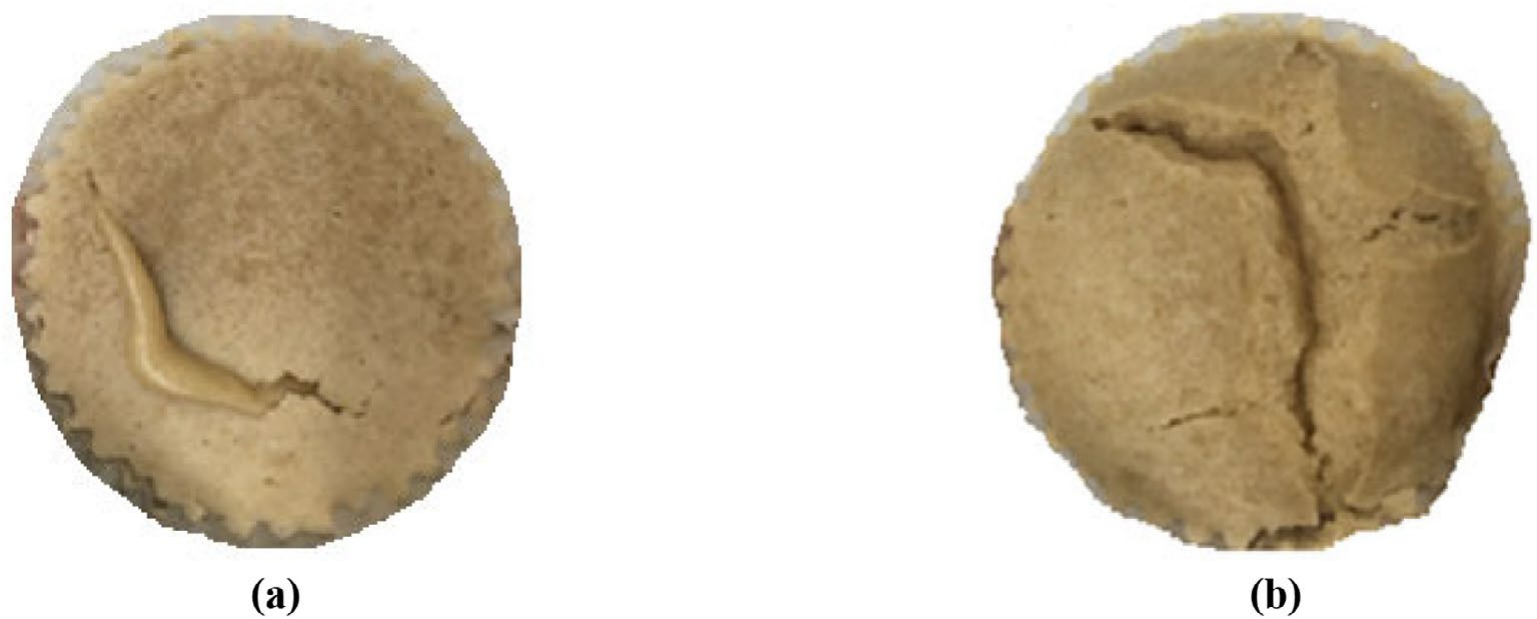
Cake surface fracture during superheated steam injection baking at 160°C for (a) 2 min and (b) 4 min.

Figure 4 shows the creation of deep fractures at the surface of the cake during superheated steam injection at 160°C. These fractures promoted the vaporization of the water located in the inner parts of the product and reduced more the moisture content of the cake (Figure 3). A contrary result was observed during bread baking which was explained by the extended degree of starch gelatinization and early formations of crust at higher temperatures (Osman et al. 2017). The lowest moisture content was related to baked cake in 180°C. María Micaela Ureta, Olivera, and Salvadori (2014) reported on how the baking temperature affected the water content in various sample areas and the amount of water lost.

### 3.3 Volume Expansion

**FIGURE 5:**
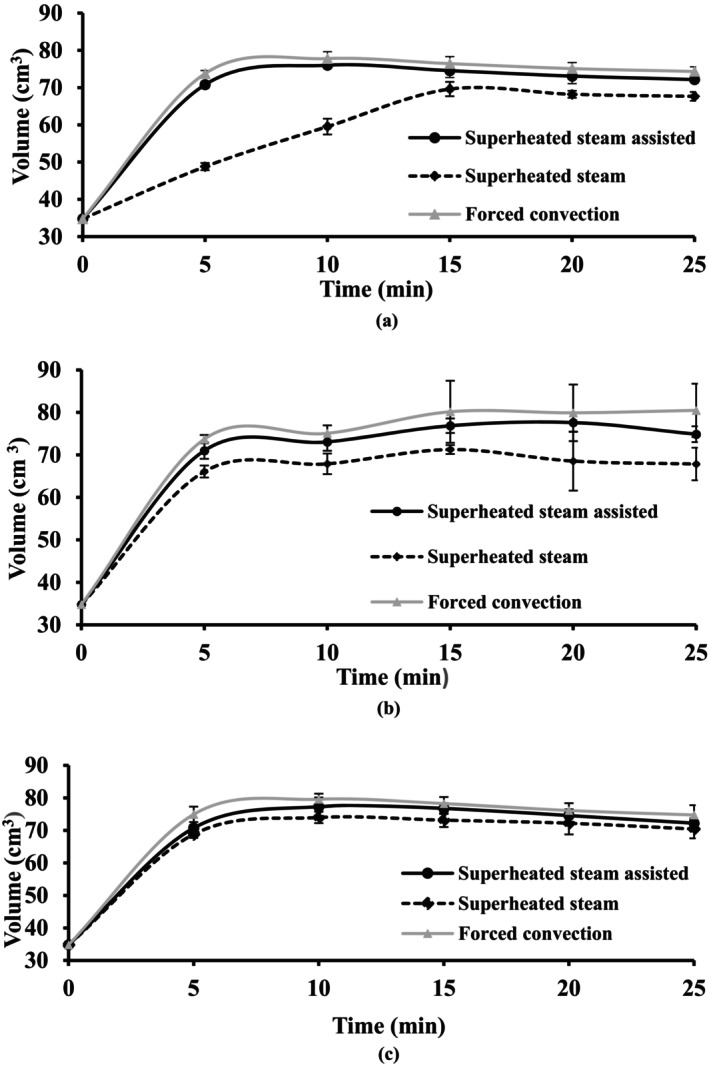
Volume expansion of the cake during baking at (a) 140°C, (b) 160°C and (c)180°C using different methods.

Volume expansion mainly took place at the early stage of the baking process (0–5 min). As shown in Figure 5, the cake volume reduced by using the superheated steam baking method, while the highest volume was obtained during a forced convection baking treatment (*p* < 0.05). At the early stages of superheated steam baking (0–5 min), a sharp loss in the moisture content of the dough surface took place resulting in a rapid formation of the crust and preventing the volume expansion; it probably was due to the changes in the compressive force equilibrium between the inside and outside of the dough. As a result, the surface cracked and increased volume is localized. Due to the fact that the temperature and air humidity in the baking chamber have a significant impact on the rate of heat transfer (Mondal and Datta 2008), the air humidity in the oven during baking is known to have a major effect on the expansion of the dough at the start of baking (Dessev et al. 2020).

A maximal rate of heat transfer is not always necessary or desirable. Altering the heating rate during baking could potentially affect the product's effective crumb structure by changing the kinetics and degree of amylopectin crystal disordering, granule swelling, and amylose leaching. These modifications would then have an effect on the textural modifications made to the product during storage (Patel, Waniska, and Seetharaman 2005).

A higher cake volume was observed at a higher temperature than 160°C (*p* < 0.05) which was in agreement with other reports (Lara et al. 2011; María Micaela Ureta, Olivera, and Salvadori 2016). However, as a result the more loss in the moisture content, baking at 180°C reduced (*p* < 0.05) the cake volume (Sani et al. 2014; Therdthai, Zhou, and Adamczak 2002). This results are in agreement with Shittu, Raji, and Sanni (2007) which were reported that baking temperature and time had an effect on physical Characterization of cassava‐wheat bread.

### 3.4 Oven Spring

**FIGURE 6:**
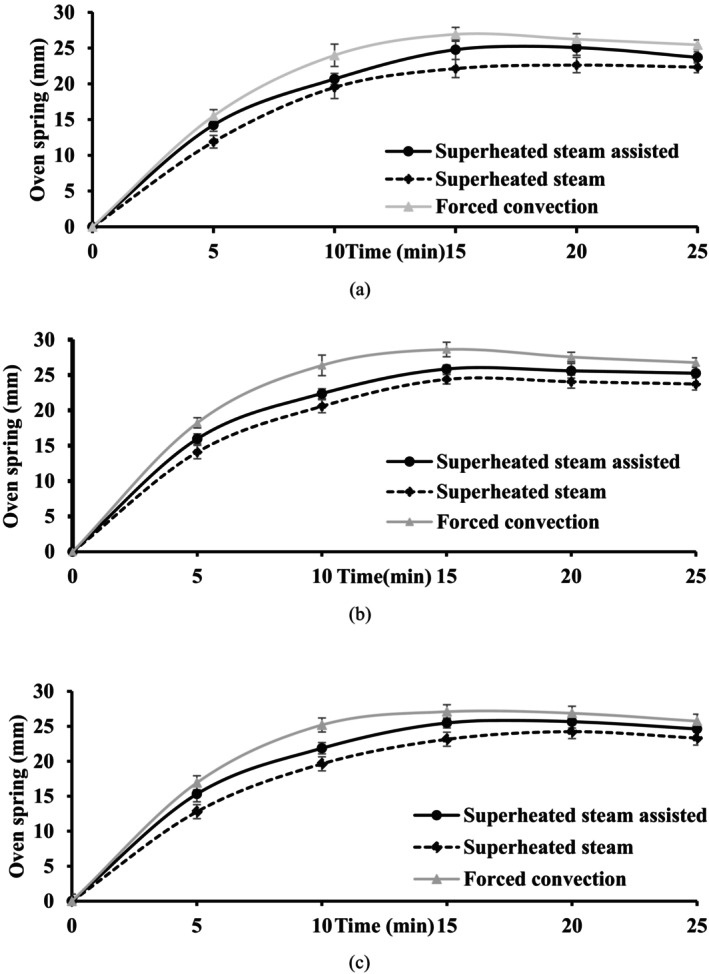
Changes in oven spring of cake during baking at (a) 140°C, (b) 160°C and (c) 180°C using different methods.

The degree of oven spring is an indicator of the crumb characteristic; as the more oven spring reflects an airy interior and little value represents a dense and compact crumb. The findings of the oven spring showed a high correlation (0.92–0.97) with the results of the volume expansion. In the first 10 min, these parameters increased rapidly and then reached a plateau. A direct relationship between the volume and oven spring has been previously reported (Bakare et al. 2014). A high oven spring was obtained in the forced convection baking process. While due to the rapid increase in the surface temperature, less oven spring was observed in the sample baked using the superheated steam‐assisted method. In all baking methods (Figure 6), the oven spring increased up to 15 min and then slightly decreased in the final stages (20 and 25 min) due to the collapse of the dough (*p* < 0.05).

This result is consistent with the findings of Li, Wang, and Krishnan (2020), who describe steamed breads as having a dense loaf with a chewy texture and a smooth, white, leathery exterior, distinguishing them from the conventional brown crust (Li, Wang, and Krishnan 2020). The rise achieved during oven spring plays a crucial role in creating a light and airy cake texture, while inadequate oven spring may lead to a dense and less appealing product. A properly risen cake not only looks more attractive but also exhibits a desirable crumb structure. Monitoring oven spring is essential for maintaining consistency in production quality. It allows bakers to evaluate the effectiveness of their ingredients, formulations, and baking conditions (e.g., temperature and humidity) (Kehinde et al. 2021).

### 3.5 Color Development

**FIGURE 7:**
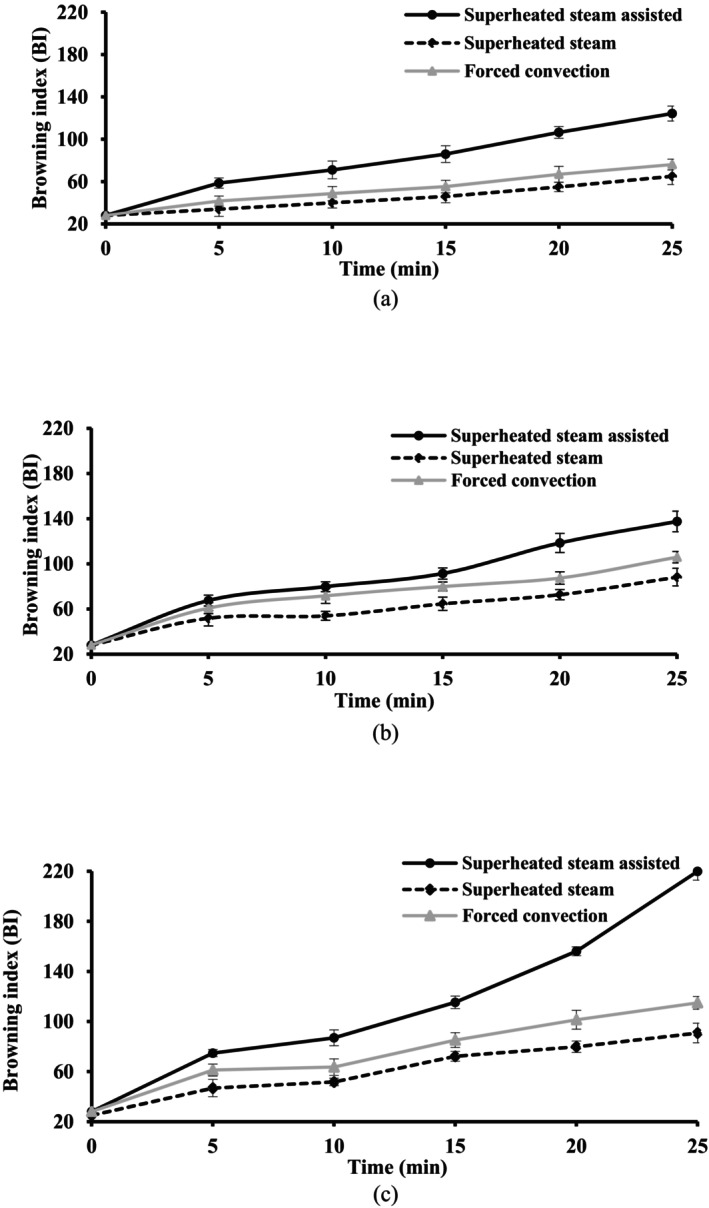
Alteration in browning index of the cake during baking at (a) 140°C, (b) 160°C and (c) 180°C using different methods.

Figure 7 shows the variation of BI, monitoring the crust color intensity, as a function of baking time at different baking method temperatures. Highest BI was observed when the products were baked in superheated steam‐assisted conditions. Superheated steam baking resulted in lower BI values (*p* < 0.05) possibly due to the more condensation of steam on the surface of the product and the lower crust temperature. It can be mentioned that there was a strong relation between BI, temperature, and time of baking. The rate of Maillard reaction improved by a considerable reduction in the moisture content of the cake surface (Petisca et al. 2013). In this regard, a high negative correlation (−0.94–0.99) was recorded between decreasing moisture content and increasing BI.

As reported in Table 3, the constant rate of BI alterations increased at a higher baking temperature (*p* < 0.05). “*k*” was in the range of 0.0022–0.0276 (min^−1^) and increased at higher temperature during the same baking method. The higher reaction rate constants were found during baking using the superheated steam‐assisted method. It showed the repressive effect of superheated steam injection on color formation. A higher temperature is required to bake the crumb fully (Figure 7). The low thermal conductivity of the cake caused a low rate of heat transfer to the center of the sample, while the temperature at the surface, particularly influenced by irradiative heat transfer from the oven walls to the cake surface. The surface became drier as the moisture evaporated and in this condition surface temperature increased towards the oven temperature. Higher water content at lower temperatures (140°C–160°C) increased the required time for developing the crust color.

**TABLE 3:** Reaction rate constants of browning index during cake baking.

Baking conditions	Temperature (°C)	Browning index	
		*K* (min^−1^)	*R* ^2^
	140	0.0033	0.98
Forced convection	160	0.0057	0.96
	180	0.0071	0.96
	140	0.0022	0.96
Superheated steam	160	0.0038	0. 95
	180	0.0059	0.97
	140	0.0064	0. 95
Superheated steam‐assisted	160	0.0178	0.97
	180	0.0276	0. 98

### 3.6 Effect of Delayed Superheated Steaming on Some Properties of Cake

**FIGURE 8:**
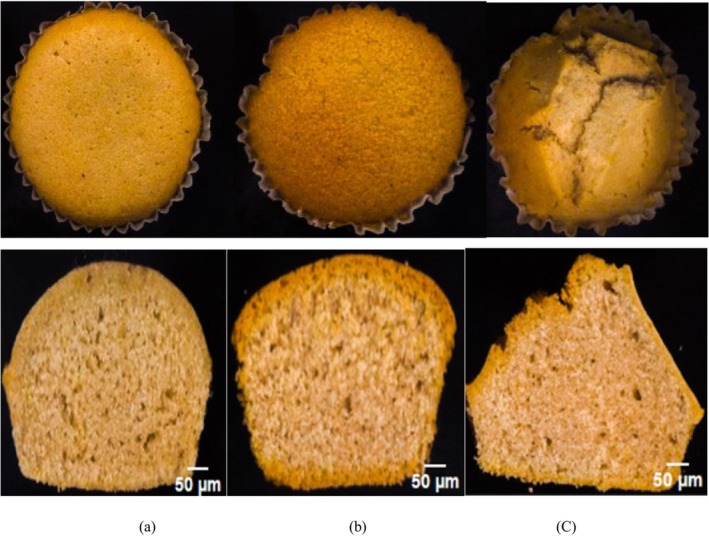
Typical cross‐sectional cake images ((a) control, (b) delayed steaming and (c) full steaming).

Large volume, attractive shape, desired moisture content, pleasant color, and aroma are important quality attributes of cakes (Al‐Dmoor 2013). The baking conditions influence significantly these quality attributes and therefore the consumer acceptance (Figure 8). Baking at higher temperatures induces high crust color and firmness and low specific volume. In agreement with Sakin‐Yilmazer et al. (2013), it was shown that crust formation takes place mainly in the early stage of baking (after 8–10 min of baking). Baking with superheated steam increases the rate of crust formation due to faster evaporation of surface moisture, decreasing the quality of the cake. Late injection of superheated steam therefore can delay this evaporation and produce a cake with higher moisture content than convention method (*p* < 0.05). Moisture content increases due to water vapor condensation at the surface of the product, decreasing the temperature of the product surface (Table 4). Delayed superheated steaming influences the volume expansion (*p* < 0.05), due to an expansion of cells. This fact produces a porous structure in the cake which decreases the firmness (*p* < 0.05). There is usually an inverse relationship between volume expansion and hardness structure (Cauvain and Young 2009). Table 4 shows, browning index increase in delayed superheated steaming in comparison force convention method (*p* < 0.05).

**TABLE 4:** Effects of baking conditions on moisture content, volume, hardness, and browning index (BI) of baked products.

Baking conditions	Moisture content (% dry basis)	Volume (m^3^)	Hardness (N)	BI
Superheated steam	0.35^a^	65^c^	4.3^c^	80^c^
Delayed superheated steam‐assisted	0.30^b^	91^a^	4.8^b^	120^a^
Forced convection	0.21^c^	75^b^	5.9^a^	110^b^

*Note:* Values with different letters (a–c) within the same column differ significantly (*p* < 0.05).

Injection of superheated steam from the beginning of the baking process has negative effects on the quality attributes of the cake due to the early formation of the crust. Therefore, limits product expansion and brings an intensive and firm structure. Delayed steaming improved some properties of cake due to steam condensation occurs on the dough resulting in a plasticization of the dough surface of the dough and larger volume expansion. In the case of condensed steam, the moisture which has condensed on the dough has provided to the surface of the product higher plasticity which facilitates its expansion during baking (Le‐Bail et al. 2011). It is an accordance to Altamirano‐Fortoul et al. (2012) that a thicker skin of the crust was observed in steamed bread.

The following advantages can generally be attained by controlling the heat transfer rate and humidity level in the baking chamber: products can be heated more effectively, and baking time can be decreased because the desired core temperature is reached quicker; production costs can be lowered; products can be heated more appropriately, and the yield can be increased; the consistency and repeatability of products can be in increased. Humidity has an impact on how well a food bakes and eventually how well it finishes (in terms of crust thickness, coloring, and crumb formation) (Dessev et al. 2020).

### 3.7 Effect of Baking Conditions on Sensory Evaluation

The overall acceptance score for superheated steam baking is much lower (2.6) compared to delayed superheated steam‐assisted baking (4.8) and forced convection (4.6), indicating a lower level of satisfaction (Table 5). Superheated steam baking receives a taste score of 2.1, suggesting that the flavor may be unappealing, potentially due to excessive moisture or uneven heat distribution. The color score is particularly low (1.5), implying that the products baked with superheated steam may lack the desirable golden‐brown crust typical of baked goods, likely due to too much moisture or uneven heat. Although the texture score is slightly better (3.2), it still falls behind compared to the other methods, possibly because superheated steam doesn't provide the crispness or structural stability needed for baked products. In contrast, delayed superheated steam‐assisted baking achieves the highest overall acceptance score (4.8), demonstrating a clear consumer preference for goods baked using this method. The taste and color scores are particularly high (4.7 and 4.9), likely due to the method's ability to retain moisture while creating a golden, appealing crust. The texture score (4.8) shows a good balance of softness and crispness, likely resulting from careful control of moisture and heat. The delayed introduction of superheated steam allows for an initial dry heat phase, promoting a better crust and internal structure before steam is added for softness. Both delayed superheated steam‐assisted and forced convection methods achieve similar high scores for taste (4.7 for both) and overall acceptance (4.8 vs. 4.6). However, delayed superheated steam‐assisted baking (4.9) excels in color compared to forced convection (4.5), likely due to the ability to balance dry heat with steam, enhancing Maillard browning without over‐drying the product. Delayed superheated steam‐assisted baking also scores slightly higher for texture (4.8 vs. 4.5), indicating that adding steam at the right time provides a softer crumb while maintaining a crisp crust. While forced convection relies solely on hot air, delayed superheated steam‐assisted baking uses controlled steam at the optimal moment, offering greater flexibility in texture and color development. In summary, superheated steam baking has significant drawbacks in taste, color, and texture, resulting in low overall acceptance. On the other hand, delayed superheated steam‐assisted baking optimizes the balance of heat and moisture, achieving the highest scores across all parameters and outperforming forced convection, particularly in color and texture. This method effectively combines the benefits of both dry and moist heat.

**TABLE 5:** Effects of baking conditions on sensory evaluation.

Baking conditions	Acceptance	Taste	Color	Texture	Total acceptance
Superheated steam	2.4 ± 0.5^a^	2.1 ± 0.4^a^	1.5 ± 0.6^a^	3.2 ± 0.5^a^	2.6 ± 0.6^a^
Delayed superheated steam‐assisted	4.8 ± 0.4^b^	4.7 ± 0.4^b^	4.9 ± 0.2^b^	4.8 ± 0.3^b^	4.8 ± 0.3^b^
Forced convection	4.7 ± 0.7^b^	4.7 ± 0.6^b^	4.5 ± 0.3^c^	4.5 ± 0.6^b^	4.6 ± 0.5^b^

*Note:* Values are expressed as mean ± SD. Values with different letters (a–c) within the same column differ significantly (*p* < 0.05).

## 4 Conclusion

This study effectively underscores the intricate relationship between baking methods, time, and temperature, and how they shape the quality of reformulated cakes. The different baking techniques—forced convection, superheated steam, and superheated steam‐assisted methods—demonstrated varied impacts on key quality attributes, such as moisture retention, volume expansion, and color development. Notably, excessive moisture in the oven atmosphere and condensation on the product surface increased the cakes' moisture content, while superheated steam accelerated crust formation, limiting volume expansion and yielding a cake color akin to unbaked products. Convection baking, with its drier atmosphere, resulted in the lowest moisture content. Among all the methods tested, delayed superheated steam injection emerged as the most promising technique, offering cakes with balanced moisture content, improved volume, a porous structure, and reduced firmness.

Future research could innovate by integrating multi‐zone baking systems and AI‐driven smart controls for precision baking. It can also explore nutrient enhancement, dynamic flavor profiling, and novel applications like sustainable ingredient use or waste valorization.

## Author Contributions

**Zohreh Mokhtari:** data curation (equal), formal analysis (equal), investigation (equal), visualization (equal), writing – original draft (equal). **Aman Mohammad Ziaiifar:** conceptualization (equal), formal analysis (equal), investigation (equal), supervision (equal), writing – review and editing (equal). **Mehran Alami:** conceptualization (equal), investigation (equal), methodology (equal). **Mahdi Kashaninejad:** conceptualization (equal), writing – review and editing (equal). **Sara Aghajanzadeh:** visualization (equal), writing – review and editing (equal). **Adel Dezyani:** investigation (equal), visualization (equal), writing – review and editing (equal). **Edris Arjeh:** formal analysis (equal), investigation (equal), writing – review and editing (equal).

## Ethics Statement

This article does not contain any studies with human or animal subjects.

## Conflicts of Interest

The authors declare no conflicts of interest.

## Data Availability

Data will be made available on request.
